# A qualitative investigation of the impact of nature walking on the health benefits achieved by older Korean immigrants living in U.S.

**DOI:** 10.1080/17482631.2025.2572508

**Published:** 2025-10-20

**Authors:** Junhyoung Kim, Su Youn Kim, Hyo Jin Ju, Areum Han

**Affiliations:** a Department of Health Behavior, School of Public Health, Texas A&M University, College Station, TX, United States; b Center for Community Health and Aging, Texas A&M University, College Station, TX, United States; c Department of Global Leisure Services, Yeungnam University College, Yeungnam University of Science and Technology, Daegu, Republic of Korea; d Department of Medical Humanities, College of Medicine, Catholic Kwandong University, Gangneung, Republic of Korea; e The Convergence Institute of Healthcare and Medical Science, Catholic Kwandong University International St. Mary’s Hospital, Incheon, Republic of Korea; f Department of Biomedical Big Data Convergence, College of Medicine, Catholic Kwandong University, Gangneung, Republic of Korea

**Keywords:** Older Korean immigrants, nature walking, qualitative research, leisure activities, psychological wellbeing, cultural bonding, physical health, health benefits

## Abstract

**Purpose:**

The purpose of this study was to investigate the health benefits associated with nature-based walking among older Korean immigrants living in the United States.

**Method:**

Semi-structured, in-depth interviews were conducted with sixteen older Korean immigrant participants. Interviews explored participant motivations for joining nature walking groups and the specific health benefits that were derived from participation in such groups. Thematic analysis was used to identify major themes in collected data.

**Results:**

Analysis revealed three primary themes: (a) improvement of psychological wellbeing that was characterized by stress reduction, emotional resilience, ad alleviation of loneliness; (b) development of cultural bonding that was demonstrated by the building of enhanced interpersonal relationships and cultural exchanges among participants; and (c) increased physical health that was indicated by improved mobility, endurance, and physical strength.

**Conclusions:**

Our findings underscore the importance of culturally sensitive leisure activities such as nature walking that significantly enhance the psychological, social, and physical health of older Korean immigrants. These insights are valuable for designing targeted health promotion interventions whose goal is to improve the quality of life and facilitate the healthy aging of this population.

## Introduction

Older Asian immigrants residing in the United States often encounter adaptation challenges associated with acculturation such as learning a new language, navigating new cultural values and beliefs, and experiencing a lack of social support and limited community-based programme engagement (Kim et al., 2019, 2020). Due to encountering these challenges, older Asian immigrants are likely to experience a poorer quality of life and lower levels of life satisfaction (Chen et al., 2021; Pan et al., 2018). One of the important factors related to improving the health and wellbeing of older Asian immigrants is the encouragement of leisure activity engagement. Research has provided evidence of the efficacy of leisure activity participation in health promotion and successful aging (Kim et al., 2014) and has provided evidence that participation in a variety of leisure activities contributes to improved physical, social, and mental health in older adults including older Asian immigrants (Kim et al., 2020).

Some studies have investigated the relationship between the level of engagement in leisure activities and the health benefits achieved by older adults (Heo & Lee, 2010; Heo et al., 2013). The findings of these studies suggest that committed engagement in certain leisure activities can lead to health benefits among older adults (Stebbins, 1992, 2001). For example, Kim et al. (2014) examined the benefits of committed engagement in sports by older Korean adults who were members of a sports club and found that participants reported gaining psychological benefits that included increased social wellbeing and improved physical health, and that engagement in the context of a sports club allowed them to gain a variety of physical, mental, and social benefits.

Among available leisure activities, walking is widely recognized as one of the most accessible and frequently practiced forms of physical activity by older adults due to its minimal skill requirement, cost-effectiveness, and ease of engagement (Han et al., 2021; Jiang & Sun, 2021; Liu et al., 2020). In particular, nature walking performed in outdoor environments such as trails, parks, and green spaces has been shown to play an important role in improving the health and wellbeing of older adults (Jiang & Sun, 2021; Liu et al., 2020). Research has provided evidence of the impact of nature walking for improving physical strength and endurance, and alleviating negative psychological symptoms (e.g., stress, depression, and loneliness) (Jones et al., 2021; Kim et al., 2020). Moreover, nature walking provides opportunities for older adults to build social cohesion and strengthen their social interactions with others (Kim et al., 2020; Kruize et al., 2019; Lee, 2020).

From the perspective of committed engagement in leisure activities, a nature walking group initiated by older Korean immigrants can be understood as a community-based activity in which participants meet regularly to engage in organized walks in natural settings. While committed engagement in leisure activities plays an important role in improving the health and wellbeing of older adults (Brown et al., 2008; Heo & Lee, 2010; Heo et al., 2013), little research has focused on the potential health benefits that can be gained through participation in such groups by older Korean immigrants, and few studies have investigated the health benefits of club or sports participation within Eastern cultural contexts (Kim et al., 2014). In addition, the limited number of studies that have investigated the health benefits gained through leisure engagement by older Korean immigrants have suggested that participation in leisure activities allowed them to reduce acculturative stress, strengthen personal relationships with other similar ethnic groups, and increase their social wellbeing (Kim & Kim, 2013; Kim et al., 2019). Building on this literature, our study explored the health benefits associated with participation in nature walking groups by older Korean immigrants.

In light of the potential benefits to be gained through nature walking for older Korean immigrants, we explored the health benefits of participation in a nature walking group by older Korean immigrants. To accomplish the goal of our study we pursued two primary research questions:

1. Why do older Korean immigrants organize and participate in nature walking groups with other Korean immigrants?
2. What benefits do older Korean immigrants gain through their participation in nature walking groups?

By addressing these questions through a qualitative lens, we have provided valuable insights into the health benefits associated with participation in nature walking groups in this population. Our findings provide critically needed data that will inform the development of culturally sensitive interventions that promote healthy aging and enhance the quality of life of older Korean immigrants. Ultimately, our goal is to contribute to the growing body of literature describing the benefits that can be derived from participation in leisure activities and health promotion activities by older Korean immigrants who actively pursue nature walking and offer actionable scientific evidence for both researchers and practitioners in the field of public health.

## Methodology

### Research design

We employed a qualitative research design with semi-structured in-depth interviews to explore the health benefits of nature walking achieved by older Korean immigrants. Crabtree and Miller (1999) stated that in-depth interviews are “a particular field research data-gathering process designed to generate narratives that focus on fairly specific research questions” (p. 93). Previous studies that captured the leisure and health benefits achieved by Korean immigrants suggested the semi-structured, in-depth interviews would be the most appropriate methodology to use to capture the detailed experiences of older Korean immigrants (Kim et al., 2019).

### Participants and data collection

A purposeful criterion sampling strategy was used for participant recruitment. Participant inclusion criteria required that participants must be over 60 years of age, an immigrant from South Korea, and have been a member of a nature walking group for more than 6 months. Our research team contacted the organizer of a nature walking group, and with his permission, we held an orientation meeting and distributed a study flyer. Interested participants contacted us via email or telephone after which our team conducted informational meetings to obtain informed consent from participants. Once agreed, we arranged an individual meeting at the convenience of the participant and conducted the interview. Pseudonyms were used to protect confidentiality. In total, 16 participants (seven males and nine females) between 63 and 90 years of age were interviewed. The sample size was determined by theory saturation from previous studies that examined the impact of leisure activity participation on the health of Korean immigrants (Kim & Kim, 2013; Kim et al., 2019). Ethical approval for this study was obtained from the Institutional Review Board of Indiana University (Approval No.: #11123). Due to the participant age group and different levels of comfort with written English, the research team provided comprehensive verbal explanations to participants about the aims, procedures, and potential risks associated with participation in the study, and their right to withdraw from the study at any point without consequence. Following these explanations, written informed consent was obtained from all participants prior to their participation. Participant privacy and confidentiality were strictly maintained throughout the study, and all consent forms were securely stored in the research records of the principal investigator.

### Interview protocol

Our research team developed an interview protocol based on previous studies (Heo et al., 2013; Kim et al., 2019) and adhered to the interview strategies proposed by Legard et al. (2003) that are based on the use of content mapping and content mining questions. Content mapping questions were created to capture overall experiences related to nature walking such as motivations. Questions included “Please tell me about your life experience in the U.S.” and “What made you interested in joining this nature walking group?” To specify the health benefits derived from their participation, content mining questions were used such as, “Based on your experiences, what role, if any, has nature walking played in helping you deal with challenges in your life?” and “How does this activity contribute to your health?” After each interview, participants completed a brief demographic questionnaire that solicited data describing participant sex, age, and educational background. All interviews were audio-recorded, transcribed verbatim in Korean and later translated into English. The complete interview protocol used in this study is provided in Appendix A.

### Data analysis

We analysed our data using the constant comparative method outlined by Creswell and Poth (2017). According to Creswell and Poth (2017, p. 126), “In the constant comparative method, the researcher simultaneously codes and analyses data in order to develop concepts; by continually comparing specific incidents in the data, the researcher refines these concepts, identifies their properties, explores their relationships to one another, and integrates them into a coherent explanatory model.” We followed McCracken (1988) five-step process that included: (a) generating each interview transcript; (b) organising and thoroughly reading each transcript; (c) creating general themes with direct quotes within each transcript; (d) comparing general themes across transcripts and extracting salient excerpts to specify sub-themes; and (e) interpreting the general and sub-themes. Each transcript was independently reviewed by each investigator, and the small number of discrepancies that were identified were resolved through discussions that resulted in consensus.

### Trustworthiness

To enhance the credibility and rigour of the data analysis, we incorporated two strategies: back translation and member checking. Due to participant language preferences, we conducted interviews in Korean, and to ensure the accuracy of translation from Korean to English, two bilingual graduate students provided back-translation. In this process, our team produced the initial English translations and then randomly selected several paragraphs from each transcript for review. The bilingual students independently compared the translated text with the original Korean passages, and any discrepancies in meaning, terminology, or nuance were discussed collaboratively until consensus was reached. No major differences emerged, which provided further assurance of the reliability and clarity of the translated data. In addition, member checking was conducted to validate our interpretations of the collected data with participants. Our research team shared some portions of main themes and interpretations with participants to allow them to review and evaluate them as proposed by Peterson et al. (2007) and rate them as “satisfactory” or “unsatisfactory.” The member checking was conducted once with each participant over the course of the study and lasted approximately 30 min. All participants rated the themes they were given as satisfactory, confirming the rigour of the data analysis.

## Findings

In this qualitative study, we identified the potential health benefits that resulted from participation in a nature walking group by older Korean immigrants. Based on the statements of participants, three main themes associated with nature walking in a group were identified: (a) improving psychological wellbeing, (b) enhancing social wellbeing, and (c) increasing physical health. These themes indicate that participation in a nature walking group with other Korean immigrants allowed them to improve their physical, mental, and social health, and may have contributed to improvement in overall health quality.

### Improving psychological wellbeing

Improving psychological wellbeing was the most salient theme that emerged from the data. Based on the reported experiences and statements of participants, three interrelated subthemes were identified: (a) stress reduction, (b) restorative connection with nature, and (c) more positive emotions and feelings.

#### Stress reduction

All participants reported feeling calm, composed, and relaxed while engaging in a nature walking group with others. They mentioned that they often experienced life challenges associated with aging and adaptation challenges such as language barriers, limited social networks, and cultural and ethnic differences. They used similar expressions: “being outdoors made me feel composed,” “it (nature walking) made me blow away my stress,” and “walking with other Korean adults reduced my life stress.” These statements indicate that nature walking in a group helped reduce stress and the impacts of life challenges. For example, Jang (female, 74) stated that she appreciated her time on the trails, indicating that nature walking gave her a meaningful start to the day and said,

*I felt alive every day and appreciated my time walking with my friends. By walking on the trails every morning, I felt more energetic and experienced a sense of positive feelings.*

Some participants who experienced significant language barriers said that walking on the trails/parks surrounded by trees relieved the stress caused by their struggles with the language. They described their walking group as a “stress-free” area where they could not only exercise but also experience minimal acculturative stress. They mentioned that speaking Korean with other nature walking participants and being surrounded by a familiar cultural community fostered feelings of safety and comfort. For example, Jung (female, 78) said, “I did not worry about speaking English and it was such a relief.” She also reported that the nature walking not only increased her level of physical activity, but also provided “stress-free exercise” with other Koreans.

#### Restorative connection with nature

Participants consistently described positive experiences associated with interacting with nature. They mentioned that local parks and trails gave them unique, meaningful places where they felt connected. By visiting to different sites, they built a unique bonding and connection with them. For example, Son (male, 82) stated, “these trails started to feel like home.” He mentioned that he picked up trash while he was walking with his walking group that gave him a sense of contributing to the community.

Many participants recalled memories about their childhood in South Korea by walking on neighbourhood trails and local parks with other club members. While walking with other members, they shared childhood routines and landscapes from South Korea. They mentioned that being outdoors led to conversation and informal storytelling that helped them share their feelings and emotions. For example, observing birds and small animals and noticing seasonal flowers allowed them to experience emotional states such as calmness, happiness, and joy. Son (male, 82) stated that he enjoyed watching animals (e.g., squirrels and deer) while walking in local parks. He also said that he remembered watching these animals when he was young in South Korea. For example, Park (male, 83) stated,

*I felt much younger when I walked in the nature… sometimes I brought some snacks to feed squirrels and it brough me joy and fun.*

He also mentioned that interacting with nature and enjoying natural surroundings (e.g., beautiful scenery) reduced his psychological distress.

#### Improved positive feelings and emotions

Most participants shared their assessments of their personal lives before and after joining the nature walking group. They consistently indicated that they spent most of their time at home watching television and using the internet before they joined the nature walking group. They also mentioned that they encountered difficulty in locating the resources that are necessary to participate in exercise programmes. Due to sedentary behaviours and limited resources for physical activity, they felt useless and lonely. Since joining the group, they believed that they had become mentally stronger and sharper, and appreciated the value of being outdoors walking.

According to Su (female, 76),

*Living in a different country has continued to be challenging for me. Due to the cultural differences and language barrier, I often preferred to stay at home and spent most of my time on the internet… Joining the walking club gave me a sense of emotional support by connecting with others. This helped me grow mentally stronger and more resilient.*

They used expressions such as “walking on the trail in the early morning helped me deal with loneliness.”

Similarly, Lee (female, 79) reported mental clarity and improved mood by being outdoors with her walking group. She stated, “my head feels much clearer after walking with my friends.” She appreciated her time outdoors and beginning her day with group walks. Although she had joined only recently, she reported that her life was much richer and her mood had improved.

These examples provide qualitative evidence that nature walking in a group can reduce stress, foster connection to nature, and improve positive feelings and emotions. It appears that these benefits are associated with improved psychological wellbeing in older Korean participants.

### Enhancing social wellbeing

Enhancing social wellbeing was another salient theme that emerged from the data. Participants reported that nature walking served as a strategy for fostering positive social connections. Two specific subthemes were identified as social wellbeing: (a) intra-group friendship and (b) social support. These subthemes resonated with gains in social health.

#### Intra-group friendship

All participants indicated that participation in the nature walking group fostered a supportive environment in which they established and developed friendships with other Korean participants. They shared similar experiences in which they encountered various cultural challenges (e.g., language barriers, limited social networks, cultural conflicts), and how joining the nature walking group allowed them to easily develop new friendships. They described nature group members as ‘family’ in which they treated each other like family members. Jang (female, 74) said,

*This group (nature walking) means a lot to me, and they were like my family. They were always there for me, and when I was sick, they stopped by my house and dropped off Korean food for my recovery*.

She further indicated that she felt comfortable with sharing her personal issues with other walking group members.

All participants mentioned that their exchange of personal information and challenges while they were engaging in nature walking was made easier by sharing their conversations in the Korean language. They discussed a variety of topics that included health issues, doctor information (bilingual doctors), housing, food, and cultural events during the walk. Through these interpersonal interactions, they believed that they had developed a unique cultural bond and group cohesion. According to Park (male, 83),

*I have been a member of this group over eight years… I did not have any friends who were similar to my age… since I joined this group, I made good friends, and we do other sports together such as golf, badminton, and table tennis.*

He also mentioned that he had initiated a bible study group for other members.

#### Social support

Most participants mentioned that they continued socialising with group members beyond walking in activities like eating brunch or drinking coffee or tea after nature walking. They stated that most members were willing to spend more time together every morning walking and believed that they received a strong sense of social support. For example, Son (male, 82) described the nature walking group as “a family” where they supported each other. They celebrated special events for each member such as birthdays and provided informational support such as health-related resources and community events. He mentioned that immigrating to a new county was often challenging, and his group offered transportation and language help for those with limited English. This unique social support system contributed to a greater sense of social community in which older Korean participants received support and attention.

Su (female, 76) reported enjoying spending more time with other members after walking. Similarly, Jung (female, 78) said,

*Since my husband passed away four years ago, I had no idea of what to do and felt lonely and depressed… without my friends (nature walking group members), I could not have a good life.*

These examples indicate that nature walking helped participants foster a unique cultural bond and form friendships with other members. By sharing similar immigration experiences, they created mutual understanding and provided emotional support.

### Increasing physical health

All participants identified higher levels of physical activity and better sleep quality as main forms of increased physical health that were related to participating in a nature walking group. They indicated that nature walking helped them to become more physically active and experience less sleep disturbance.

#### Increased physical activity

Considering nature walking as a form of physical activity, all participants shared their experience that nature walking helped them become more physically active and mentioned that walking in nature every morning became their routine physical activity that resulted in improved physical health. Many participants observed that walking in natural surroundings enhanced their mobility, endurance, and overall strength, and some participants who had experienced previous mobility issues mentioned that continuous participation in nature walking helped them improve their mobility. Han (male, 75) mentioned that his joints felt better after regular nature walking exercise and that he felt more energetic since he started walking in nature. Other participants shared similar experiences and reported gaining greater stamina and positive energy throughout the day. According to Jang (female, 74),

“I felt that my legs were getting stronger, and I was gradually able to walk longer distances. I did not feel tired as fast.”

Most participants believed that walking in nature motivated them to be more physically active compared to walking on treadmills at the gym or in Korean senior centres. They were more willing to go outside for a walk and not only exercised, but also enjoyed their interactions with nature and appreciated the opportunity to be physically active in nature. Jung (female, 78) said that she preferred to walk in natural surroundings over walking on the treadmill because she felt bored at the gym. Similarly, Lee (female, 79) stated,

*I really enjoyed walking on the soft soil of the trail, it helped reduced my chronic pain… being in nature always gave me extra energy and stamina, and I never felt discouraged to walk with my team members.*

She also mentioned that nature walking was a sustainable form of physical activity, and that she gained physical benefits in many ways.

Some participants who used wearable technology to measure how many walking steps they took were encouraged to set their personal goals with other participants. They mentioned that they gradually increased their walking steps with other participants and celebrated their achievements. Such personal goal setting helped them become more physical active, and that they believed they had gained physical strength. Song (male, 73) shared that he enjoyed keeping track of his walking steps because it motivated him to become more physically active and allowed him to gradually increase his step count and walk longer distances. He said,

*As I mentioned earlier, I did not know what to do after my retirement when I became physically inactive. After joining the walking club, I started going outdoors more often, became physically active, and was able to maintain good stamina.*

#### Better sleep quality

Some participants who experienced sleep disturbance noted that they had experienced better sleep quality after joining the walking group. They mentioned that they fell asleep more easily and woke less often. Han (male, 75) said, “after morning walking, I slept through the night without walking.” Due to improved sleep quality, he believed that he gradually walked longer distances and maintained his stamina over time. Similarly, Su (female, 76) shared similar experiences in which she had experienced some difficulties in sleeping at night and mentioned that her sleep had improved after the nature walking every morning.

Participants noted that improved sleep contributed to better performance in everyday activities and that they felt less fatigued and experienced higher levels of energy and stamina. For example, they mentioned that they sustained efforts across household chores, errands, and longer walks. According to Jung (female, 78),

“*At first, when I joined the walking group, I felt very tired and napped after I got back. After a few weeks, I felt more energy, and I believed that my body needed some time to adjust the morning walks… nowadays, I feel more independent with the household chores, and I feel like a new person with much more physical energy.”*

These statements provide evidence of the importance of nature walking as a form of physical activity, and how participants believed that nature walking was beneficial for their physical health by contributing to higher levels of physical activity and better sleep quality.

## Discussion

We explored the health benefits that can be derived from participation in nature walking by older Korean immigrants. The findings of our study provide suggestive evidence that participation in a nature walking group can lead to physical, mental, and social health benefits for older Korean immigrants, and that participation in such a group allowed participants to maintain their cultural values and beliefs and develop unique intergroup friendships with other Korean immigrants. The findings of our study suggest that participation in walking club activities can play an important role in improving the health and wellbeing of participants.

Previous studies have provided evidence that nature walking can provide a variety of health benefits for older adult participants (Jiang & Sun, 2021; Jones et al., 2021; Kim et al., 2020; Liu et al., 2020), and that older adults improved their health and life satisfaction through participation in exercise in natural settings. The findings of our study are aligned with the findings of prior studies that found that nature walking can improve mental health by reducing stress and psychological challenges, develop friendships, and improve physical functioning. Our findings expand the body of knowledge describing the effects of nature walking on health and provide evidence of the impact of participation in nature walking groups on the health of older Korean immigrants.

While adaptation challenges can impact the ability of older immigrants to participate in physical leisure activities, a small number of studies (Kim & Kim, 2013; Kim et al., 2016) have provided evidence that leisure activity participation provides opportunities to reduce acculturative stress, enhance intergroup friendships and strengthen the resilience of older Korean immigrants. Our study also captured the cultural benefits related to older Korean immigrant adults participating in a nature walking group with other Korean immigrants who shared similar immigration experiences. The results of our data analysis show that participants experienced a sense of belonging and connectedness with other Korean immigrants that resulted in building culturally specific social networks. This finding reinforces the importance of culturally related activity groups for immigrant groups, especially those who have experienced cultural adaptation challenges.

Substantial evidence has been presented on the physical and mental health benefits that can be achieved by older adults related to participation in leisure walking (Han et al., 2021; Jiang & Sun, 2021), and many studies have provided evidence that older adults who frequently engaged in leisure walking were likely to report higher levels of physical and mental health (Han et al., 2021; Liu et al., 2020). The findings of our study support the idea of the importance of nature walking as a catalyst for increasing physical functioning. In addition, while previous studies did not specify what types of leisure walking were evaluated, the findings of our study suggest that walking in natural surroundings can be instrumental in improving the health and wellbeing of older Korean immigrants. This finding highlights the importance of nature walking as a form of health promotion.

The findings of our study also provide evidence of the potential that a variety of technologies possess to encourage and sustain activity participation as several of the study participants used wearable technologies to monitor their walking steps. A growing body of literature has provided evidence of the benefits of wearable technology for the health promotion and physical activity participation of older adults (Mercer et al., 2016). The findings of our study suggest that older immigrants who used a variety of technologies can gain health benefits such as active engagement in walking and activity motivation, a finding that suggests the potential of the use of technological applications as a method to increase the health and wellbeing of older adults.

Researchers have suggested that place attachment develops through dynamic interactions between individuals and their surrounding environments that result in affective, emotional, and functional bonds with specific locations or settings (Low & Altman, 1992; Williams & Roggenbuck, 1989). Consistent with this perspective, the findings of our study indicate that older Korean participants exhibited a strong emotional attachment to neighbourhood parks and trails. This attachment was expressed not only through their engagement in nature-based walking activities but also through recollections of childhood routines and landscapes from South Korea. Such experiences may contribute to a sense of belonging and enhanced memory recall. Importantly, these results suggest that place attachment may extend its influence into cognitive domains, as the opportunity to connect with nature and reminisce about past events and activities can support memory processes and potentially enhance the cognitive functioning of older adults.

The findings of our study are subject to several limitations that should be addressed in future studies. First, we employed qualitative methodology to explore the health benefits associated with participation in a nature walking group by older Korean immigrants that may limit the generalisability of the findings. Future mixed methods studies should be conducted to investigate the health benefits of participation in nature walking groups by older immigrants. Second, there are individual factors (e.g., acculturation levels, time since immigration, socio-economic status, physical limitations related to aging, frequency of participation) that may affect participation in nature walking and the health benefits gained by older Korean immigrants. It would be beneficial if future studies investigated the relationships between these variables to produce a comprehensive overview of the health-related benefits of nature walking in groups. Third, as there are other Asian immigrant populations who organize their own groups for physical activity participation, broadening the representation of a wide range of Asian immigrant populations may provide deeper, more comprehensive insights into how to design culturally appropriate programmes that are effective for other Asian immigrant populations. Lastly, while we employed two key strategies (back translation and member checking) to improve the rigour and credibility of our data analysis, additional formal triangulation methods (the use of multiple coders, systematic comparison of interview data with observational data) could further strengthen the validity of future data analyses. Future researchers should consider incorporating multiple coders, integrating field notes, and/or comparing interview data with other qualitative or quantitative data sources to enhance methodological rigour.

## Conclusion and implications

Our study provides a preliminary qualitative investigation into how participation in a Korean immigrant nature walking exercise group helped participants improve their health and wellbeing. The findings of our study underscore that the health benefits that can be derived from participation in nature walking groups by older Korean that include improved physical, social, and mental health. The findings of our study also suggest the importance of the availability of culturally appropriate exercise programmes in which older immigrants can pursue leisure activity participation. In addition, several participants indicated that they were motivated by the use of technologies such as step counters or mobile phones that monitored their walking activity. These devices and others can play an important role in helping older immigrants remain physically active and gain health benefits. While participation in nature walking groups provides opportunities to build emotional and social support, integrating technology into their exercise routines may increase and sustain their engagement and ultimately contribute to greater physical, mental, and social health benefits. Further, stakeholders such as Korean community centres, municipal Parks and Recreation departments, and other immigrant-serving organisations are encouraged to co-design nature-based exercise programmes and develop translated, culturally tailored materials to support participation. These types of collaborations can create the accessible pathways that are critically important to older immigrants who wish to engage in active leisure and improve their health.

## Data Availability

The data supporting the findings of this study are available from the corresponding author upon reasonable request. Due to ethical and participant privacy considerations, study data will not be made publicly available.

## Appendix A

### Interview protocol

The following protocol guided the semi-structured interviews conducted in this study. It was developed based on previous studies (Heo et al., 2013; Kim et al., 2019) and the interview strategies outlined by Legard et al. (2003). The protocol included both content mapping questions (to capture broad experiences and motivations) and content mining questions (to explore health benefits in depth).

### Grand-tour questions

• “Do you feel most comfortable speaking in English or Korean?”
• “Please tell me a little about yourself.”
• “How long have you been participating in the nature walking programme?”

### Mini-tour questions

• “Why do you participate in the nature walking programme?”
• “What benefits have you experienced from participating in this group?”
• “Based on your experiences, what role, if any, has nature walking played in helping you deal with challenges in your life as an immigrant?”
• “Have you experienced any challenges in seeking to participate or while participating in the nature walking programme? If so, could you describe them?”

### Conclusion

“Thank you for your time and contribution. The information you have shared is very helpful, and I appreciate your willingness to speak with me. Would you like me to keep you informed of the findings from this study?”

## References

[cit0001] Brown, C. A., McGuire, F. A., & Voelkl, J. E. (2008). The link between successful aging and serious leisure. *International Journal of Aging and Human Development*, *66*(1), 73–95. 10.2190/AG.66.1.d 18429484

[cit0002] Chen, Y., Wang, Z., Dong, W., Xu, J. H. C., Wu, S. J., Zhang, X., & Chen, C. (2021). The pathways from perceived discrimination to self-rated health among the Chinese diaspora during the COVID-19 pandemic: Investigation of the roles of depression, anxiety, and social support. *International Journal for Equity in Health*, *20*(1), 192. 10.1186/s12939-021-01537-9 34454508 PMC8401352

[cit0003] Crabtree, B. F., & Miller, W. L. (1999). *Designing qualitative research*. Sage.

[cit0004] Creswell, J. W., & Poth, C. N. (2017). *Qualitative inquiry and research design: Choosing among five approaches* (4th ed.). SAGE Publications.

[cit0005] Han, A., Kim, J., & Kim, J. (2021). A study of leisure walking intensity levels on mental health and health perception of older adults. *Gerontology and Geriatric Medicine*, *7*, 1–8. 10.1177/2333721421999316 PMC7923965 33718525

[cit0006] Heo, J., Culp, B., Yamada, N., & Won, Y. (2013). Promoting successful aging through competitive sports participation: Insights from older adults. *Qualitative Health Research*, *23*(1), 105–113. 10.1177/1049732312457247 22927703

[cit0007] Heo, J., & Lee, Y. (2010). Serious leisure, health perception, dispositional optimism, and life satisfaction among senior games participants. *Educational Gerontology*, *36*(2), 112–126. 10.1080/03601270903058523

[cit0008] Jiang, Y., & Sun, H. (2021). Exploring the characteristics and influencing factors of leisure walking based on the demand of behavior. *Sustainability*, *13*(8), 4105. 10.3390/su13084105

[cit0009] Jones, K. O., Lopes, S. S., Kelly, C., Welsh, R. S., Chen, L., Wilson, M., & Shi, L. (2021). A qualitative study on participants’ experiences with a community-based mindful walking intervention and mobile device activity measurement. *Complementary Therapies in Medicine*, *57*, 102640. 10.1016/j.ctim.2020.102640 33388390

[cit0010] Kim, B., Jun, H., Lee, J., & Kim, Y. M. (2020). Social support, activities of daily living, and depression among older Japanese and Koreans immigrants in the US. *Social Work in Public Health*, *35*(4), 163–176. 10.1080/19371918.2020.1761922 32364061

[cit0011] Kim, J., & Kim, H. (2013). The experience of acculturative stress-related growth from immigrants’ perspectives. *International Journal of Qualitative Studies on Health and Well-being*, *8*(1), 1–11. 10.3402/qhw.v8i0.21355 PMC3784669 24070225

[cit0012] Kim, J., Kim, J., & Han, A. (2020). Leisure time physical activity mediates the relationship between neighborhood social cohesion and mental health among older adults. *Journal of Applied Gerontology*, *39*(3), 292–300. 10.1177/0733464819859199 31271089

[cit0013] Kim, J., Moon, S., & Song, J. (2016). Is leisure beneficial for older Korean immigrants? An interpretative phenomenological analysis. *International Journal of Qualitative Studies on Health and Well-being*, *11*(1), 33103. 10.3402/qhw.v11.33103 27914195 PMC5134826

[cit0014] Kim, J., Suh, Y. I., & Kim, J. (2019). Identifying leisure constraints associated with acculturation among older Korean immigrants. *International Journal of Qualitative Studies on Health and Well-Being*, *14*(1), 1–8. 10.1080/17482631.2019.1655378 PMC6719260 31452458

[cit0015] Kim, J., Yamada, N., Heo, J., & Han, A. (2014). Health benefits of serious involvement in leisure activities among older Korean adults. *International Journal of Qualitative Studies on Health and Well-being*, *9*(1), 1–9. 10.3402/qhw.v9.24616 PMC4110381 25059979

[cit0016] Kruize, H., van Der Vliet, N., Staatsen, B., Bell, R., Chiabai, A., Muiños, G., & Stegeman, I. (2019). Urban green space: Creating a triple win for environmental sustainability, health, and health equity through behavior change. *International Journal of Environmental Research and Public Health*, *16*(22), 4403. 10.3390/ijerph16224403 31717956 PMC6888177

[cit0017] Lee, S. (2020). Perceived neighborhood environment associated with older adults’ walking and positive affect: Results from the health and retirement study. *Journal of Aging and Physical Activity*, *29*(3), 536–543. 10.1123/japa.2020-0236 33333489

[cit0018] Legard, R., Keegan, J., & Ward, K. (2003). In-depth interviews. In J. Ritchie, & J. Lewis (Eds.), *Qualitative research practice: A guide for social science students and researchers* (pp. 138–169). Sage.

[cit0019] Liu, Z., Kemperman, A., & Timmermans, H. (2020). Correlates of older adults’ walking trip duration. *Journal of Transport Health*, *18*, 100889. 10.1016/j.jth.2020.100889

[cit0020] Low, S. M., & Altman, I. (1992). Place attachment: A conceptual inquiry. Human Behavior & Environment: Advances in Theory & Research, 12, 1–12.

[cit0021] McCracken, G. D. (1988). *The long interview* (Vol. 13). Sage.

[cit0022] Mercer, K., Giangregorio, L., Schneider, E., Chilana, P., Li, M., & Grindrod, K. (2016). Acceptance of commercially available wearable activity trackers among adults aged over 50 and with chronic illness: a mixed-methods evaluation. *JMIR mHealth and uHealth*, *4*(1), e4225. 10.2196/mhealth.4225 PMC4749845 26818775

[cit0023] Pan, X., Chahal, J. K., & Ward, R. M. (2018). Quality of urban life among older adults in the world major metropolises: A cross-cultural comparative study. *Ageing Society*, *38*(10), 1991–2013. 10.1017/S0144686X17000234

[cit0024] Peterson, W. E., Sword, W., Charles, C., & DiCenso, A. (2007). Adolescents' perceptions of inpatient postpartum nursing care. *Qualitative Health Research*, *17*(2), 201–212. 10.1177/1049732306297414 17220391

[cit0025] Stebbins, R. A. (1992). *Amateurs, professionals, and serious leisure*. McGill-Queen's Press-MQUP.

[cit0026] Stebbins, R. A. (2001). Serious leisure. *Society*, *38*(4), 53–57. 10.1007/s12115-001-1023-8

[cit0027] Williams, D. R., & Roggenbuck, J. W. (1989). Measuring place attachment: Some preliminary results (Abstract), Proceedings of the National Recreation and Parks Association Symposium on Leisure Research, 1989. San Antonio, TX (October, 1989).

